# Dr. Reynell Coates’ Contributions to Surgery

**Published:** 1842-04-30

**Authors:** Reynell Coates


### MEDICAL EXAMINER.

NEW SERIES.

No. 18.] PHILADELPHIA, APRIL 30, 1842. [Vol. I.

### *Contributions to Surgery.*

BY REYNELL COATES, M. D.

### *Note on the means of avoiding Excoriations of the Heel and Perineum in Fractures of the Lower Extremities, treated by Permanent Extension.*

Continued from p. 262.

In the preceding part of this paper, I have endeavoured to estimate the value of the simple buckskin gaiter as a means of permanent extension. Two different measures have been recommended and practised by Dr. Joseph Hartshorne, to prevent the occurrence of excoriation from the rugosity of the leather, which is almost sure to be produced by the continued action of the tapes. Firstly; the substitution of a padded sock for the simple gaiter; and, secondly; the direct application,—beneath the band,—of a strip or strips of buckskin spread with adhesive plaster, in order to prevent the immediate action of the extending force upon the skin of the patient.

With regard to the first of these measures, it is only necessary to remark that it does not, in any degree, relieve the difficulty resulting from the natural extensibility of buckskin, and superadds upon it the compressibility of the wadding or stuffing. This last, being usually composed of cotton, increases the facility with which the apparatus yields to the tonic contraction of the muscles now no longer resisted by their natural antagonist, the entire bone. In addition to this evil, the thickness of the sock renders it utterly impossible to arrive at an available approximation to accurate admeasurement between the superior anterior spinous process of the os ilium and the lowest point of one of the malleoli,—the only possible mode in which the surgeon can assure himself of the real effect of his extension. Moreover, the use of the padded sock by no means secures, with certainty, that equality or uniformity of pressure over the instep and tendo Achillis which is one of the principal indications in the treatment. This latter fact will be illustrated hereafter by a note of an interesting case. These grounds appear to me sufficient to warrant the condemnation of the padded sock.

The second measure—the interposition of buckskin spread with adhesive plaster between the extending band and the skin—is liable to far more serious objection. Whenever a substance perfectly impervious to moisture is applied closely upon the cuticle, the escape of the insensible perspiration of the part is prevented; it is condensed beneath the dressing; the cuticle swells and, finally, becomes detached in flakes; the cutis vera, under the perpetual action of moisture and in the absence of light, becomes rapidly changed in structure, assuming, more and more nearly, the characters of mucous membrane with its high susceptibility to irritation. Causes which would prove altogether innoxious under other circumstances, often give rise to very troublesome excoriation or ulceration in parts long habituated to the action of such impervious dressings. Nor is this all: the ingredients of which adhesive plaster is composed are directly irritating in their nature, and, the cuticle being once removed, prove fertile sources of inflammation. When there exists an epidemic or endemic tendency to erysipelas, hospital gangrene, puerperal fever, or their congeners, the continued use of adhesive plaster is always especially dangerous.* This branch of the subject may be dismissed with the remark that I have never seen a single instance in which this method of diminishing or equalizing pressure was employed in fractures without the production of ulcerations, whether the application was made to the ankle or to the perineum.

Next in order comes the folded handkerchief, mentioned in my last communication. Silk is an application scarcely less bland than buckskin, and it is very nearly inextensible. We are perfectly sure that we can retain any advantage gained in the elongation of the limb, by means of a material so unyielding; but this, also, has its disadvantages. By its bulk, the handkerchief somewhat interferes with the admeasurement; and, as it is inevitably thrown into folds which act sharply and unequally upon the skin, unless the band be rolled with more than common care, a certain amount of irritation is usually produced by its application. A careful surgeon will always double the handkerchief diagonally in the first instance, and then involve it regularly from the apex of the triangle thus formed towards its base, and in such a manner as to make the band about two and a half or three inches wide in the middle.

When this extending band is employed, it will be found that it yields to a certain extent in the first instance, in consequence of the bias of the thread resulting from the peculiar mode of folding; and, even after the first application, the dressing yields a little to the retractile force of the muscles, permitting slight shortening of the limb in most fractures of the thigh ; but in fractures of the tibia, the broader surfaces in apposition usually prevent this accident. Silk fibres, however, being very slightly extensile, the band very soon assumes its permanent length, under the action of the extending force, and the contractions of the muscles cease to produce any appreciable shortening after the second examination of the limb. It will be perceived that the objections urged against the handkerchief are far less important than those which bear upon the buckskin garter, or the padded sock. With care in folding, it constitutes, perhaps, the best of the ordinary extending bands. If it should give rise to irritation by inequality of action, this is easily checked by enveloping the ankle and instep in a gaiter of buckskin without tapes,—as a mere protection,—and using the handkerchief over it. The real difficulties in the way of its success are its bulk, which interferes with accurate measurement as well as the precision and security of the knot by which it is secured to the splint or foot-bar of the apparatus.

There are two modes of employing the handkerchief, and each requires attention to peculiar precautions. In fractures of the femur, it is customary to apply it in the following manner. The middle of the band is placed over

* This principle will assist in explaining the evils resulting from the too long continuance of oleaginous dressings in wounds and ulcers, so well known to surgeons, though condemned by Mr. Liston in the introductory chapters of his Practical Surgery, in a sweeping manner which I have ventured to censure in a recent notice of that work, (*Examiner*, p. 200.) Many days are usually required to produce an important change in the cuticle by such means;—as is seen when we employ India rubber sheets for the cure of chronic rheumatism;—and the more immediate ill effects of oily dressings are generally due to the oxydation of the material, which renders it directly irritating.

the tendo Achillis just above the malleoli; the ends are brought forwards, over and above the malleoli; they are then simply crossed directly in front of the articulation of the ankle,—not on the instep; they are next carried directly downwards over the sides of the foot, immediately in front of the malleoli; they are then tied very loosely, and by a single knot, beneath the sole, and the free ends are finally secured to the apparatus of extension. By changing the position of the knot beneath the sole, so as to make it approximate the inner or outer edge of the foot, we can determine to a considerable extent the degree of abduction or adduction of the foot; but of this more will be said hereafter.

One of the unavoidable evils attendant on this mode of application, consists in the line of direction of the extending force being somewhat anterior to the axis of the limb, though parallel with it. Any mechanist will perceive immediately that this circumstance inevitably produces a tendency to force the heel backwards when the band is tightened; and pressure upon the heel is one of the most important causes of pain and difficulty in the treatment of fractures of the lower extremities in the extended position. There is also an obvious loss of power resulting from duplex operation of the band, and an increased extending force is therefore requisite. But, when properly adjusted, this action of the band is very slight, for the direction of force is not very far removed from the precise axis of the limb: in the treatment of uncomplicated fractures of the femur it may be safely neglected, but not so when the bones of the leg are implicated. In these cases the general hyperemia and the excessive excitability of the limb are very often extended to the neighbourhood of the ankle-joint, and invade the parts which are subjected to the direct pressure of the band: consequently; any increase of force under these circumstances is much more prejudicial. Again; the heel participating in the same irritable condition, the mere weight of the limb not unfrequently occasions great uneasiness to the patient; as will be seen by reference to the recent clinical reports of the Pennsylvania Hospital, forwarded to this journal by Dr. Edward Hartshorne. If, to these remarks, we add that the backward tendency of the heel demands considerable care to prevent a forward angular deformity at the seat of fracture,—a danger which is greater, like the other difficulties just noticed, in proportion to the proximity of the injury to the ankle-joint—we shall perceive that this application of the handkerchief, though sufficiently convenient in most fractures of the thigh, is of questionable propriety in those of the leg. Indeed; when the injury is very near the malleoli, or when a solution of continuity in the os femoris is complicated with contusion, laceration, or fracture very near the ankle-joint, it is wholly inadmissible for obvious reasons.

To meet the last named difficulty, another method of employing the handkerchief has been used in the Pennsylvania Hospital, in cases requiring permanent extension, where the ankle-joint or its neighbouring soft parts are implicated. This method, I believe, was introduced by Dr. John Rhea Barton. It is somewhat difficult of description without the aid of a graphic illustration, but if the reader will practice literally on his own person the following directions, I believe he will be able to comprehend the manner of application.

Take a silk handkerchief folded in the manner already described. Double it so as to form two tails, one of which shall be six inches longer than the other. Place the point of duplicature over the internal malleolus of the injured limb, and extend the band so that the shorter tail shall point exactly forwards and the longer tail backwards, parallel with the mesial plane. Carry the shorter tail obliquely towards the external malleolus, anteriorly ; so as to pass immediately below it, covering the anterior part of the astragalus ; and then pass it perpendicularly downwards, (or towards the foot of the bed) on the outer side of the tarsus, holding it somewhat tense in that direction. Carry the longer tail towards the external malleolus, posteriorly; so as to cover that portion of the fibula; and extend the remainder of the tail directly towards the toes, making it cross over the descending portion of the shorter tail, keeping it always reasonably tense. In the next place, make this long end to descend obliquely over and in front of the shorter extremity ; wind it half round the latter, in front or on the side next the toes ; carry it thence inwards beneath the sole of the foot; let it rise on the inner side of the tarsus; pass it between the internal malleolus and the handkerchief, at the place of commencement ; and then revert the remainder of the tail perpendicularly downward, along the inside of the tarsus. The extremities of the two tails, now ranging parallel to each other below the foot, should next be tied in a single knot, and their extremities are then available for attachment to the splints or cross piece upon which extension is effected.

The action of this band, when properly applied, is usually in the exact direction of the axis of the limb ; but, by a little care in adjusting the position of the crossings of the tails at the inner and outer malleoli, the bandage may be made to elevate or depress the heel at pleasure ; and, by a similar adjustment of the knot beneath the sole,—carrying it a little towards one or the other edge of the foot,—any required degree of abduction or adduction is readily secured.

The moment the band thus arranged is put upon the stretch, the lateral crossings descend to the level of the sole, or below it, leaving the malleoli entirely bare. We then find one portion of the handkerchief covering the anterior part of the astragalus and the scaphoid bone, and descending with a very slight obliquity backwards to the crossings directly beneath the axis of motion in the ankle; another portion embraces the heel, immediately above the posterior projection of the os calcis, and passes obliquely forward toward the same crossings. The remaining portion lies beneath the foot, and furnishes the direct means of extension.

No part of this very beautiful bandage comes in contact with any portion of the leg. It is confined to the foot, and exerts its pressure chiefly on the extreme upper part of the instep and the heel. A little lateral pressure on the side of the foot occasionally produces irritation there, and requires the interposition of a piece of buckskin or pellets of soft cotton ; but even this is very rarely required, if the looseness of the dressing permit the lateral crossings to descend a little below the sole, and if the lower knot be placed at least two inches from the foot. These precautions should be generally observed. In extremely irritable cases it is sometimes advisable to omit the lower knot altogether, employing the extremities of the bandage as simple tapes. This knot formed no part of the apparatus as contrived by Dr. Barton, but was introduced by myself because it gives us the power of regulating the adduction of the foot—a matter of so much consequence in injuries near the ankle, that it far outweighs the evil of a slight increase of the lateral pressure.

The great advantages of this method of using the handkerchief are the absence of all embarrassment in admeasurement or in dressing wounds near the ankle during the continued action of the extending force, and the perfect command of the position of the foot. I have even used it, *very gently ap-*

*plied*, as a sling to secure the latter, in one case of compound fracture within two inches of the inner malleolus. The only disadvantage of the method consists in the intervention of another rather feeble and extensible joint between the fracture and the extending band. When the first mode of using the handkerchief is adopted, we extend mainly from the leg; the ankle joint being scarcely put upon the stretch. If much permanent force be employed upon the latter, stiffness, irritation of the joint, and sometimes in young subjects, weakness of the ligaments may be produced.

Let us conclude this branch of our subject by the conclusions at which I have arrived in relation to the practical claims of the handkerchief.

The first method of application is preferable in most cases of fractures of the thigh, where considerable contractile forces must be resisted; and when adroitly managed it furnishes better means of extension than the usual gaiters and socks; being less likely to produce excoriation or ulceration. But it is proper to remark here that when the band is crossed a very little too far down upon the instep, it tends forcibly to depress the toes, producing great pain in the ankle joint, and requiring that the metatarsus should be supported by a sling attached to one of the splints on cross pieces. This necessity increases enormously the force required in extension, and renders excoriation almost inevitable. Those surgeons who delay the complete reduction of the limb too long will find that, in the endeavour to avoid the evil just mentioned, they increase the circular pressure of the band where it surrounds the leg, to the great embarrassment of the circulation. Hence follow frequent mischiefs chargeable not to the *use*, but to the *abuse* of the method.

The second method should not be employed in cases where the disposition to shortening is very strong, but it is admirably adapted to the treatment of those cases of fracture of the leg which require permanent extension, whether complicated with fracture of the thigh or not. When the injury occurs very near the ankle, it is our only available resource; and as the tendency to shortening in the most oblique fractures of the leg is resisted with comparative ease, it may be considered preferable to any other extending band in these accidents generally. I have never known it to produce excoriation, when thus employed, though the lateral pressure has produced considerable uneasiness in a few instances.

(*To be continued.*)

